# Molecular locks and keys: the role of small molecules in phytohormone research

**DOI:** 10.3389/fpls.2014.00709

**Published:** 2014-12-17

**Authors:** Sandra Fonseca, Abel Rosado, John Vaughan-Hirsch, Anthony Bishopp, Andrea Chini

**Affiliations:** ^1^Departamento de Genética Molecular de Plantas, Centro Nacional de Biotecnología- Consejo Superior de Investigaciones CientíficasMadrid, Spain; ^2^The Botany Department, University of British ColumbiaVancouver, BC, Canada; ^3^Centre for Plant Integrative Biology, University of NottinghamNottingham, UK

**Keywords:** phytohormones, chemical genomics, hormone perception and signaling, hormone crosstalk, plant chemical biology, jasmonates, agonist and antagonist, small molecules

## Abstract

Plant adaptation, growth and development rely on the integration of many environmental and endogenous signals that collectively determine the overall plant phenotypic plasticity. Plant signaling molecules, also known as phytohormones, are fundamental to this process. These molecules act at low concentrations and regulate multiple aspects of plant fitness and development via complex signaling networks. By its nature, phytohormone research lies at the interface between chemistry and biology. Classically, the scientific community has always used synthetic phytohormones and analogs to study hormone functions and responses. However, recent advances in synthetic and combinational chemistry, have allowed a new field, plant chemical biology, to emerge and this has provided a powerful tool with which to study phytohormone function. Plant chemical biology is helping to address some of the most enduring questions in phytohormone research such as: Are there still undiscovered plant hormones? How can we identify novel signaling molecules? How can plants activate specific hormone responses in a tissue-specific manner? How can we modulate hormone responses in one developmental context without inducing detrimental effects on other processes? The chemical genomics approaches rely on the identification of small molecules modulating different biological processes and have recently identified active forms of plant hormones and molecules regulating many aspects of hormone synthesis, transport and response. We envision that the field of chemical genomics will continue to provide novel molecules able to elucidate specific aspects of hormone-mediated mechanisms. In addition, compounds blocking specific responses could uncover how complex biological responses are regulated. As we gain information about such compounds we can design small alterations to the chemical structure to further alter specificity, enhance affinity or modulate the activity of these compounds.

## Introduction

### From phenotypes to molecules: early chemical genomics approaches

Plant growth, development and adaptation to the environment require the integration of many environmental and endogenous signals that, together with the intrinsic genetic program, determine overall plant responses. In this context, signaling molecules and growth regulators, collectively known as phytohormones, act as central hubs for the integration of complex environmental and cellular signals. Phytohormones such as auxins, cytokinins (CK), gibberellins (GAs), abscisic acid (ABA), ethylene (ET), brassinosteroids (BRs) salicylic acid (SA), jasmonates (JAs), and strigolactones act at low concentrations and, either alone or in combination with other hormones, regulate multiple aspects of plant development, defense and adaptation. The search for both synthetic plant hormones and hormone mimics with increased stability/activity has been central to the development of the agrochemical industry and the “green revolution” in the past century (Brown et al., 2014). Initially, organic chemists used chemically synthesized hormonal derivatives to identify novel compounds mimicking or reversing the phenotypes induced by endogenous phytohormones. For example, the discovery of the structure of the naturally occurring auxin phytohormone indole-3-acetic acid (IAA) allowed chemical synthesis of a wide array of analogs and derivatives, and phenotypic screens. These approaches identified molecules such as 1-naphthaleneacetic acid (1-NAA), 2,4-dichlorophenoxyacetic acid (2,4-D), and 2-methyl-4-chlorophenoxyacetic acid (MCPA) that are still widely used today as growth promoters or herbicides (Overbeek and Vélez, 1946; Grossmann, 2010) (Supplemental Table 1). Similarly, phenotypic screens of functional analogs of the endogenous salicylic acid signals identified compounds such as benzothiadiazole (BTH) and 2,6-dichloroisonicotinic acid (INA) that were employed in the field to enhance plant disease resistance (Conrath et al., 1995; Görlach et al., 1996; Lawton et al., 1996) (Table 1 and Supplemental Table 1).

**Table 1:** **List of molecules described in this review including molecular targets, biological activity and references**.

**Common name**	**Target**	**Biological activity**	**References**
**AUXIN**			
Gravacin	PGP19	Strong inhibitor of root and shoot gravitropism	Rojas-Pierce et al., 2007
L-kynurenin	TAA1/TARs	Inhibitor of auxin synthesis and of ethylene responses	He et al., 2011
BUM	ABCB/MBR/PGP efflux carriers	Selective inhibitor of ABCB efflux carriers. Allows discrimination with PIN	Kim et al., 2010
Alcoxy-auxins	Auxin transporters PIN, ABCB and AUX	Selective inhibitors of auxin transport. Not recognized by the receptors	Tsuda et al., 2011
α-Alkyl auxins	TIR1	Rationally designed auxin agonists and antagonists	Hayashi et al., 2008
Auxinole	TIR1/AFBs	Rationally designed auxin antagonist	Hayashi et al., 2012
Picloram	AFB5	Picolinate auxin. Agonist of auxin signaling	Walsh and Chang, 2006; Calderón Villalobos et al., 2012
IAA-Trp, JA-Trp	Unknown	Innhibitors of several auxin mediated responses	Staswick, 2009
**GIBBERELIN**			
GA3—Fluorescein	GID1 receptor	Florescent GA mimetics recognized by the receptor	Shani et al., 2013
**CYTOKININ**			
Phe-Ade	CKX and AHK3 and CRE1/AHK4 receptors	Week binding to cytokinin AHK3 and AHK4 receptors and inhibition of Cytokinin Oxidase/dehydrogenase (CKX) on cytokinin degradation	Motte et al., 2013
S-4893	CRE1 receptor	Non-competitive cytokinin antagonist by targeting CRE1 receptor	Arata et al., 2010
SS-6772 and S-4607	CRE1 receptor	CRE1 antagonists	Arata et al., 2010
**ABA**			
Pyrabactin	PYR1 and PYL1	Affects seed germination by interacting with a sub-set of PYR/PYL/RCAR ABA receptors	Park et al., 2009; Okamoto et al., 2013
Quinabactin	PYR1, PYL1-3,4	Stomatal closure. Interacts with a sub-set of ABA receptors	Okamoto et al., 2013
*ASn*	PYR/PYL	ABA antagonists. Block the interaction PYR/PYL–PP2C	Takeuchi et al., 2014
**JASMONIC ACID**			
Coronatine	COI1/JAZs	Produced by Pseudomonas syringae, is a potent agonist of JA. Binds the receptor complex	Xie et al., 1998; Katsir et al., 2008; Fonseca et al., 2009b
Vernolic acid	AOC2	Inhibits AOC2 and limits OPDA production by 50%. Affects JA synthesis	Hofmann et al., 2006
Phenidone	LOX	Animal LOX inhibition. Little effect on JA biosynthesis	Engelberth, 2011
PACOR, PAJAIle	COI1/JAZ1	Biotin-tagged photoaffinity labeled molecules that promote COI1/JAZ interaction	Yan et al., 2009a
JM-8686	AOS	Strong inhibitor of AOS activity	Oh et al., 2006
Jarin-1	JAR1	Inhibits the last step of JA-Ile biosynthesis	Meesters et al., 2104
(+)-7-*iso*-JA-L-Ile	COI1/JAZs	Endogenous jasmonate recognized by the receptor	Fonseca et al., 2009b; Sheard et al., 2010
(+)-JA-L-Ile	COI1/JAZs	Synthetic agonist of the endogenous (+)-7-iso-JA-Ile	Fonseca et al., 2009b
COR-MO	COI1/JAZs	Coronatine rational designed antagonist. Blocks JA and COR perception	Monte et al., 2014
Fluorescent jasmonate	Unknown	Migrates in tomato	Liu et al., 2012; Liu and Sang, 2013
Bestatin	Unknown	Inhibitor of aminopeptidases. Mutants insensitive to bestatin render alleles of *myc2* and *med25*	Schaller et al., 1995; Zheng et al., 2006; Chen et al., 2012
**BRASSINOSTEROID**			
Brassinazole	Cytochromes P450 DWF4 and CPD	Inhibits BR biosynthesis	Asami et al., 2000, 2001
Fluorescent castasterone	BRI1	Bioactive fluorescent labeled BR, recognized by the receptor BRI1	Irani et al., 2012
Bikinin	GSK3-like kinases, BIN2 included	Induces constitutive BR-related phenotypes by inhibiting GSK3 kinases	De Rybel et al., 2009
Brassinopride	Unknown	Inhibitor of BR action. Acts on BR synthesis and activates ethylene responses	Gendron et al., 2008
**STRIGOLACTONES**			
GR24	MAX2/DAD2/D14	A potent synthetic strigolactone analog	Gomez-Roldan et al., 2008; Umehara et al., 2008
Karrikin - KAR2	MAX2/KAI2	Generated in the smoke, structurally similar to strigolactones. Inducers of germination	Nelson et al., 2011; Hamiaux et al., 2012; Waters et al., 2012; Guo et al., 2013
Cotylimides	Unknown	Strigolactones agonist in germination, hypocothyl development and cotyledon bleeching. Revealed a crosstalk between strigolactones and light	Tsuchiya et al., 2010
**SALICYLIC ACID**			
BTH (benzothiadiazole)	Unknown	Inducer of SA-mediated defense responses, enhancing plant disease resistance in the field	Görlach et al., 1996; Lawton et al., 1996
INA	Unknown	Inducer of SA-mediated defense responses, enhancing plant disease resistance in the field	Conrath et al., 1995
Imprimatins	Two SA glucosyltransferases (SGT)	Activator of endogenous SA accumulation by blocking SA turnover. Enhancers of pathogen activated cell death	Noutoshi et al., 2012

A complementary chemical approach for the identification of bioactive molecules mimicking the activity of endogenous hormones can be based on the analysis of plant-interacting organisms. This approach revolves around organisms that have evolved the capability to produce phytohormones or phytohormone mimics to enhance disease susceptibility and counteract plant defenses. For example, characterization of the fungal pathogen *Gibberella fujikuroi* [responsible for the *bakanae* disease in rice (Kurosawa, 1926)] allowed the identification of gibberellic acid derived phytohormones (Shimada et al., 2008; Robert-Seilaniantz et al., 2011), and analysis of the bacterium *Pseudomonas syringae pv. Tomato* was instrumental for the identification of the phytotoxin coronatine (COR) (Feys et al., 1994). This is a jasmonate functional analog that the bacteria use to hijack the plant defense signaling network (Kloek et al., 2001; Brooks et al., 2004; Gimenez-Ibanez and Solano, 2013; Xin and He, 2013) (Table 1 and Supplemental Table 1).

Despite the profound contribution of those early chemical approaches in phytohormone research, these methodologies had two important limitations. Firstly, the success of these approaches relies on the serendipity of identifying a structurally amenable product from a relatively small number of natural sources. Secondly, the large collections of hormonal derivatives frequently lack chirality and their structural diversity is limited to variations in attachments within a restricted number of common skeletons (Brown et al., 2014). Therefore, these approaches only cover a small fraction of the structural possibilities present within the chemical space, and therefore reduce their potential versatility.

### From molecules to function: plant chemical biology in the genetic era

Recent decades have seen the development of a whole host of molecular and genetic tools as well as the release of complete genome sequences. Therefore, genetic strategies such as the isolation of mutations that confer altered hormonal responses and the identification of the downstream target genes have substituted the early chemical approaches and quickly became the preferred methods to elucidate the molecular mechanisms underlying phytohormone action. These genetic approaches have significantly enhanced our understanding of the molecular basis of phytohormone action (for review see Browse, 2009). In spite of its success, the use of well-established genetic tools (such as large collections of knockout and activation tagged mutants) for the identification of components in plant hormonal networks has now reached such a stage that it is becoming increasingly challenging to identify the remaining components. This recalcitrant to genetic approaches is largely due to a combination of gene redundancy, where multiple genes regulate the same process and individual knockouts have no discernable phenotype, and gene lethality, which prevents the identification of loss-of-function mutations in essential genes (Robert et al., 2009; Fernández-Calvo et al., 2011; Acosta et al., 2013).

**Figure 1:**
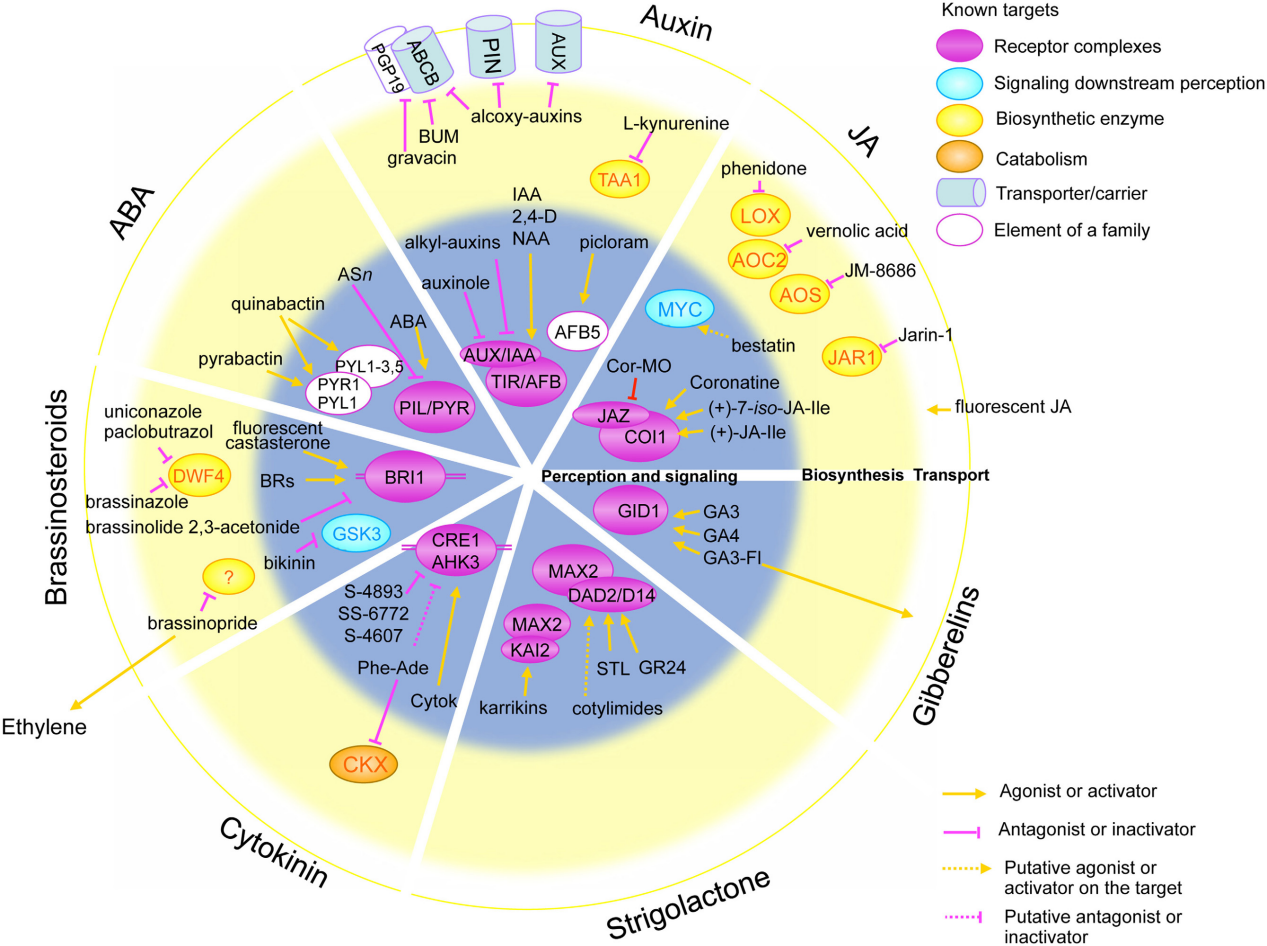
**Schematic representation of the molecular targets of small molecules acting in different hormonal pathways**. Concentric circles in the background represent the distinct biological processes in hormonal pathways: perception and signaling (gray inner circle), biosynthesis (yellow middle circle) and transport (white outer circle). Circles are divided in quadrants for distinct hormones, from the top clockwise: auxin, jasmonic acid (JA), gibberellins, strigolactones, cytokinins, brassinosteroids and abscisic acid (ABA). Ovals represent the molecular targets: receptor complexes (violet), signaling components (blue), biosynthetic enzyme (yellow) and catabolic enzymes (orange). Cylindrical shapes represent transporters and carriers. Molecules acting as activators are represented with an orange arrow toward their targets, whereas pink blocked arrows highlight antagonists and inhibitor molecules.

Fortunately, the development of genetic tools has gone hand in hand with advances in combinatorial synthesis. These advances have enabled access to highly diverse chemical libraries containing both wider spectra of molecular shapes and range of biological targets than traditional combinatorial libraries (Schreiber, 2000; Hicks and Raikhel, 2012). These chemical libraries are being used to overcome many of the limitations of purely genetic approaches. They can be used to address genetic redundancy, as small molecules are capable of modulating the active sites of whole classes of protein targets. They can also address gene lethality, as small molecules can enable the temporal and spatial blockage of specific hormonal responses in a reversible manner (McCourt and Desveaux, 2010; Tóth and van der Hoorn, 2010; Hicks and Raikhel, 2012). Hence, in the last two decades agrochemical biased libraries have been widely used in combined genetic and chemical screens aimed at the dissection of multiple physiological processes in plants. These screens have yielded valuable bioactive compounds such as gravacin (Rojas-Pierce et al., 2007), morlin (DeBolt et al., 2007), sortins (Zouhar et al., 2004; Rosado et al., 2011), hypostatin (Zhao et al., 2007), and endosidins (Robert et al., 2008; Drakakaki et al., 2011) (Figure 1 and Table 1). All these compounds are currently used to modify the activity of individual proteins or protein families in a tuneable, reversible and spatial-temporal controlled manner.

We now know that in some cases the mechanisms for perceiving individual hormones are conserved, and the same recognition systems are able to mediate response to several hormones, while in other cases unique perception strategies have evolved for individual molecules (Tan et al., 2007; Murase et al., 2008; Shimada et al., 2008; Park et al., 2009; Lumba et al., 2010; Sheard et al., 2010; Kumari and van der Hoorn, 2011). A paradigmatic example of a conserved recognition system is the “molecular glue” mechanism, first described for the auxin receptor complex, in which the auxin molecule promotes the formation of its receptor complex (Tan et al., 2007; Mockaitis and Estelle, 2008). The F-box TIR1 (TRANSPORT INHIBITOR RESPONSE 1) or AFBs (AUXIN SIGNALING F-BOX) cannot bind, or bind at very low affinity, auxin without the interaction of the co-receptors Aux/IAA (AUXIN RESISTANT/INDOLE-3-ACETIC ACID INDUCIBLE) and the inositol hexakisphosphate (IP6) cofactor. Only the structural modifications produced by the formation of the tetrameter stabilize the hormone perception. The same mechanism also occurs in jasmonate perception, since the hormone induces the formation of the receptor tetramer complex formed by JA-Ile, the F-box COI1 (CORONATINE INSENSITIVE1), the co-receptor JAZ (JASMONATE ZIM DOMAIN PROTEIN) and the inositol pentakisphosphate (IP5) cofactor (Chini et al., 2007; Thines et al., 2007; Sheard et al., 2010). Gibberellins are also sensed by a similar perception system: active GAs promote the establishment of the complex formed by GID1 (GIBBERELLIN INSENSITIVE DWARF1) receptor and the F-box SLY1 (SLEEPY1) (Murase et al., 2008; Shimada et al., 2008). In contrast, other phytohormones are perceived by specific protein complex based on different recognition systems. For example, the PYR1 (PYRABACTIN RESISTANT 1) and PYL (PYRABACTIN RESISTANT-LIKE) receptors bind ABA directly in cooperation with the co-receptors type 2C protein phosphatases, such as ABI1 (ABA INSENSITIVE 1) and ABI2 (ABA INSENSITIVE 2). The subsequent inactivation of the phosphatases induces the SNF1-type kinase activity, which in turn regulates ABA-dependent gene expression and downstream signaling cascades (Weiner et al., 2010). CK perception and signal transduction pathway occur through a phosphorelay similar to bacterial two-component response systems. Briefly, CK binds directly to the membrane-located HISTIDINE KINASE (AHK) receptors. This initiates a phosphorelay cascade where a phosphoryl group is translocated via the HISTIDINE-CONTAINING PHOSPHOTRANSFER PROTEINS (AHPs) and then to the RESPONSE REGULATOR (ARRs) transcription factors. Type-B ARRs regulate the transcription of cytokinin responsive genes and type-A ARRs acting as negative feedback regulators to desensitize plants to excess cytokinin (Kieber and Schaller, 2014).

The discovery of each of these hormonal response mechanisms has enabled the implementation of innovative chemical genomics approaches, and the rational design of chemical tools for phytohormone studies. These will be described in details within this review.

### From function to targets: screening for novel proteins/complex using screens and tagged-molecules

Bioactive chemicals identified from forward or reverse chemical screens are very useful for the dissection of complex biological processes. One advantage of this technique is that it can either target specific proteins or multiple members of redundant gene families. However, the identification of the cognate biochemical target/s remains a very complex process that depends on the type and affinity of the chemical-target interaction, as well as the abundance of the target sites (Robert et al., 2009). Throughout the years, researchers have performed diverse genetic screens in *Arabidopsis thaliana* for resistance to specific chemicals. These have allowed the subsequent biochemical and genetic identification of cognate targets. For example, chemical genetic screens for resistance to bikinin (an activator of the brassinosteroids responses) showed that it could bind directly and inhibit a subset of the GSK3 (GLYCOGEN SYNTHASE KINASE 3) kinase family (De Rybel et al., 2009) (Table 1 and Supplemental Table 1). Similarly, a screen for resistance to gravacin (a strong inhibitor of root and shoot gravitropism) identified the auxin efflux transporter PGP19 (P-GLYCOPROTEIN 19) as its molecular target (Rojas-Pierce et al., 2007) (Table 1 and Supplemental Table 1).

These genetic-based approaches require further validation of the target since mutations can prevent the drug from reaching the site of action due to either metabolic alterations or uptake/translocation defects. As an alternative, different biochemical tools have been developed for target identification (Kolb and Sharpless, 2003). These include collections of “tagged” chemical libraries that possess reactive moieties permitting the immobilization of active compounds through “click chemistry.” Although there are several potential click reactions, the Copper (I) catalyzed synthesis of triazoles from azides and acetylenes has become the standard for the generation of “click libraries” and the chemical species in those libraries possess an amine handle that enables affinity resin synthesis via reaction with activated carboxylic acid affinity resins (Kolb et al., 2001). For example, a library of tagged molecules was used in a high throughput approach to detect active proteins in the whole proteome of *Arabidopsis thaliana* (Van der Hoorn et al., 2011). Additionally these compounds can also contain a fluorophore to enable visualization of hits in living cells or other contexts.

In the following sections of this review we will describe recent landmark chemical genomics approaches and place special emphasis on their roles in the elucidation of the molecular mechanisms underlying hormonal regulation, considering all stages from the biosynthesis to the perception of the signal.

## Phytohormone homeostasis

Different endogenous and environmental stimuli regulate the tissue-specific biosynthesis of phytohormones. The synthesis and catabolism of these molecules are tightly regulated as they are very bio-active. For example at least three, partially redundant, biosynthetic pathways have been identified so far for synthesizing auxin (Stepanova et al., 2008; Zhao, 2008). The complete biosynthetic network is not yet fully understood. However, the use of auxin analogs played an important role in identifying many of the mutants impaired in auxin biosynthesis. For example, the *tir2* (*transport inhibitor response 2*) mutant was isolated as an NPA (1-N-naphthylphthalamic acid)-resistant mutant and subsequently shown to encode TAA1 (TRYPTOPHAN AMINOTRANSFERASE OF ARABIDOPSIS 1), one of the key enzymes in the indole-3-pyruvic acid (IPA) auxin biosynthetic pathway (Yamada et al., 2009). However, mutants with modestly perturbed levels in auxin show strong pleiotropic effects and this restrict their usefulness in investigating specific aspects of auxin action. In addition, the enzymes that mediate key biosynthetic steps are often redundant and require the generation of higher order combinations of mutants to detect observable phenotypes. Therefore, the identification of compounds enabling the manipulation or blockage of specific biosynthetic pathways is invaluable. An example of such compound is the recently isolated inhibitor of the auxin biosynthesis L-kynurenine (He et al., 2011) (Figure 1, Table 1 and Supplemental Table 1).

L-kynurenine (Kyn) was originally identified as an inhibitor of the ethylene-induced auxin biosynthesis in roots (He et al., 2011) (Figure 1). Subsequently, He and colleagues demonstrated that Kyn is an alternate substrate for auxin biosynthetic enzymes TAA1/TAR (TRYPTOPHAN AMINOTRANSFERASE RELATED) and that it competitively inhibits TAA1/TAR activity (Stepanova et al., 2008). Strikingly, Kyn binds to the substrate pocket of TAA1/TAR proteins in a highly effective and selective manner, but does not bind to other aminotransferases. The use of Kyn has overcome some genetic limitations of traditional approaches, such as the sterility and lethality of double and triple mutants in the redundant *TAA1/TAR* gene family, and has enabled the blockage of auxin biosynthesis in specific tissues or plant stages (Stepanova et al., 2008). Kyn has added value to classic genetic studies. For example, by combining Kyn treatments with mutants impaired in auxin biosynthesis, it was recently shown that root-based auxin biosynthesis is required in addition to polar auxin transport to correctly pattern the root xylem axis (Ursache et al., 2014). Most enzymes within the auxin biosynthetic network are well conserved between plant species, including mosses and lichens (Finet and Jaillais, 2012). Consequently, molecules such as kynurenine that inhibit auxin biosynthesis could easily be used on other species, providing a wide range of possible applications.

Many molecules regulate the complex signaling networks responsible for plant defense, however salicylic acid plays a central role in restricting the activity of biotrophic pathogens. Genetic screens for mutants with enhanced disease resistance have mainly uncovered dwarf mutants with elevated SA levels (Murray et al., 2002; Shirano et al., 2002; Grant et al., 2003; Zhang et al., 2003; Vlot et al., 2009). To avoid the pleiotropic effects of plants with altered SA levels, researchers have long sought-after compounds enabling the manipulation of SA in a tuneable and reversible manner. Recently, a high-throughput chemical genomic screen identified the imprimatin family of molecules as enhancers of pathogen-activated cell death (Noutoshi et al., 2012). Imprimatin treatments induce the accumulation of SA, reduce its inactive metabolite SA-glucoside, and enhance plant disease resistance (Table 1 and Supplemental Table 1). Further analyses have shown that imprimatins block SA turnover through specific inhibition of two SA GLUCOSYLTRANSFERASES (SAGTs). Double knockout mutants of these SAGTs are semi-dwarf plants that consistently showed the same SA-accumulation and enhanced disease resistance as imprimatin-treated plants (Noutoshi et al., 2012). Imprimatins offer an exciting way in which synthetic compounds that can be applied to different plant species to trigger accumulation of the active endogenous SA and overcome the pleiotropic effects associated with constitutive high levels of SA. Besides the biotechnological applications, these molecules can also be used in phytohormone research to induce the accumulation of endogenous SA transiently at specific plant developmental stages, avoiding the need to use of semi-dwarf mutant lines in the redundant *SAG* genes.

Cytokinins have been long known to regulate cell division, differentiation as well as many aspects of plant development—including root growth, root/shoot branching architecture and vascular development (Werner and Schmülling, 2009; Hwang et al., 2012). Cytokinins are adenine derivatives, and the incorporation of specific side chains at the N6-position triggers their recognition as ligands for specific receptors or substrates for enzymes regulating their homeostasis. One key group of enzymes catalyzing the oxidative removal of the side chain and thereby degrading cytokinins are the CYTOKININ OXIDASE/DEHYDROGENASE (CKX) family. In *Arabidopsis* there are 7 members of the CKX family, and each has subtly different substrate specificity (Kowalska et al., 2010). A recent high-throughput chemical screen based on *in-vitro* cytokinin-dependent shoot regeneration (Motte et al., 2013) identified one novel compound, Phe-Ade (N-phenyl-9H-purin-6-amine) (Figure 1, Table 1 and Supplemental Table 1). Further biochemical studies showed that Phe-Ade induces the accumulation of endogenous cytokinin by acting as a competitive inhibitor of the cytokinin-degrading CKX enzymes and preventing cytokinin catabolism.

Brassinosteroid biosynthesis is regulated by a complex network of three redundant pathways that convert the common precursor campesterol into the active BRs. The BR biosynthetic pathways requires the activity of the cytochrome P450 DWARF 4 (DWF4), a key rate limiting P450 monooxygenase that acts on multiple intermediates in the BR biosynthesis pathways (Asami et al., 2000, 2001; Chung and Choe, 2013) and represents an ideal target to bypass the redundancy of the BR biosynthesis pathways. Uniconazole and paclobutrazol are both inhibitors of P450 monooxygenases that act as weak BR inhibitors and are able to induce accumulation of the precursor campesterol (Asami and Yoshida, 1999) (Figure 1). Subsequent analysis into the structure-activity relationship identified brassinazole (BRZ) as strong inhibitor of BR biosynthesis blocking the cytochrome P450 monooxygenase DWF4 and therefore preventing the hydroxylation of BR precursors (Asami and Yoshida, 1999; Asami et al., 2000) (Table 1 and Supplemental Table 1). BRZ was subsequently used for a genetic screen to isolate BRZ insensitive mutants. *bzr1-1D* (*brassinazole resistant 1*) and *bes1* (*bri1-ems-suppressor 1*) mutants respectively showed insensitivity to BRZ and enhanced constitutive BR responses. The phenotypes of these mutants were later shown to be caused by the stabilization of the transcription factors BZR1 (BRASSINAZOLE RESISTANT 1) and BES1/BZR2 (BRI1-EMS-SUPPRESSOR 1/BRASSINAZOLE RESISTANT 1) (Wang et al., 2002; Yin et al., 2005a). BZR1 and BES1/BZR2 are the fundamental activators of the BR signaling pathway, which regulate the expression of most BR responsive genes (Vert and Chory, 2006). The use of BZR is exemplary of the potential of integrating chemical genomics with classical genetics to identify key regulators of a hormone signaling pathway.

Jasmonic acid-isoleucine (JA-Ile) is an end product of the oxylipin biosynthetic pathway and, together with additional oxylipin molecules, it mediates several developmental processes and stress responses (Fonseca et al., 2009a; Wasternack and Hause, 2013). The oxylipin biosynthetic pathway is well understood and several inhibitors of key steps in this pathway have been reported (Wasternack and Hause, 2013). The JA-Ile biosynthesis is believed to start with the conversion of free α-linolenic acid by 13-lipoxygenases. Therefore, several general inhibitors of animal lipoxygenases (such as phenidone, aspirin, ibuprofen and ursolic acid) were tested in plants; however, they show only with limited inhibitory effects on oxylipin biosynthesis in plants, possibly due to functional redundancy or differences between animal and plant lipoxygenases (Wasternack, 1993; Farmer, 1995; Engelberth, 2011).

The subsequent biosynthetic step is catalyzed by the ALLENE OXYDE SYNTHASE (AOS) and ALLENE OXYDE CYCLASE 2 (AOC2), that mediate a non-redundant, coupled reaction producing the first cyclic oxylipin 12-oxo-phytodienoic acid (OPDA). The complete loss of cyclic oxylipins in *aos1* mutant generates sterile plants and confirmed the essential role of AOS (Park et al., 2002; Wasternack and Hause, 2013). Therefore, AOS and AOC2 represent ideal targets to inhibit the whole cyclic oxylipin pathway. Vernolic acid is a naturally occurring oxylipin first described as competitive inhibitor of the AOC of maize by Hamberg and Fahlstadius (1990). More recently, the crystal structure of AOC2 determined the direct binding of the competitive JA inhibitor vernolic acid within the AOC2 hydrophobic barrel cavity (Hofmann et al., 2006) (Table 1 and Supplemental Table 1). Biochemical assays also demonstrated that vernolic acid inhibited approximately 50% of the AOC2-mediated production of OPDA *in vitro*. In addition, the imidazole derivative JM-8686 was designed to inhibit the activity of AOS, most likely by direct binding of the imidazole group of JM-8686 to the heme iron of AOS (Oh et al., 2006). However, the subsequent use of vernolic acid and JM-8686 was very limited because the residual activity of AOS/AOC2 coupled reaction can produce enough cyclic oxylipin to mediate most plant responses.

The final step of the biosynthetic pathway is performed by JASMONOYL-L-ISOLEUCINE SYNTHETASE (JAR1), that synthesizes the bioactive hormone (+)-*7-iso*-JA-Ile by conjugating JA with the amino acid isoleucine (Staswick and Tiryaki, 2004; Fonseca et al., 2009a). Very recently, Meesters et al. reported jarin-1 as the first small molecule inhibitor of jasmonate responses identified in a chemical screen (Meesters et al., 2104). Jarin-1 inhibits many JA-mediated responses *in planta*, but did not affect reactions induced by JA-Ile, suggesting an inhibitory activity on the JA-Ile producing enzyme JAR1. Further biochemical data confirmed that jarin-1 impairs the JA-Ile synthesis and inhibits the activity of JAR1, whereas closely related enzymes are not affected. Molecular modeling suggests a direct jarin-1 binding to the active site of JAR1. Overall, jarin-1 is the first direct, specific inhibitor of JAR1 (Meesters et al., 2104) (Figure 1, Table 1 and Supplemental Table 1).

## Phytohormone transport

In plants, most hormones are mobile molecules whose inter or intra-cellular transport is required for function and control of physiological responses. With the textbook exception of auxin polar transport, the molecular mechanisms and components of hormone transport are still relatively unknown. In the case of auxins, genetics and chemistry both played essential roles in identifying and characterizing the three families of auxin transporters, AUX1/LAX (AUXIN RESISTANT 1/LIKE AUXIN RESISTANT), ABCB/MDR/PGP (ATP-BINDING CASSETTE subfamily B/ MULTIDRUG RESISTANCE/ P-GLYCOPROTEIN) and PINs (PIN-FORMED). For example, the *aux1* mutant was isolated through exploring the permeability differences between the membrane-permeable auxin 1-NAA and the membrane-impermeable auxin analog 2,4-D (Figure 1, Table 1 and Supplemental Table 1). The *aux1* mutant was discovered through its agravitropic phenotype that could only be rescued by 1-NAA (Bennett et al., 1996). AUX1 was subsequently characterized as the first IAA influx carrier (Marchant et al., 1999; Swarup et al., 2001; Yang et al., 2006). Some members of the proteins ABCB/MDR/PGP transporters have been identified as proteins with binding affinity to the auxin transport inhibitor 1- naphthylphthalamic acid (NPA) (Noh et al., 2001; Robert and Friml, 2009; Ma and Robert, 2014) (Figure 1). The initial identification of the PIN family of auxin efflux carriers occurred through the genetic isolation of the *pin1* mutant, which shows a phenotype resembling that caused by the pharmacological inhibition of polar auxin transport (Okada et al., 1991; Gälweiler, 1998).

Recently, a chemical genomic screen based on phenotyping a suite of morphological traits such as growth rate and flowering time identified a novel and potent inhibitor of ABCB efflux carriers, BUM (2-[4-(diethylamino)-2-hydroxybenzoyl]benzoic acid). BUM directly binds and inhibits ABCBs, although ABCB1 appears to be the primary target. This binding occurs without directly affecting PIN transporters, and therefore allows the specific analysis discriminating between PIN and ABCB efflux systems (Kim et al., 2010).

Auxin perception allows regulation of the intracellular accumulation of endogenous auxins by modifying the localization of several transporters. As a consequence, it is often difficult to uncouple auxin perception from auxin transport. To overcome this limitation, rationally designed molecules such as alkoxy-IAA derivates (alkoxy-auxins) were developed that specifically target auxin transporters (Tsuda et al., 2011) (Figure 1, Table 1 and Supplemental Table 1). Structural modeling testing the docking of alkoxy-auxins to the TIR1-Aux/IAA receptor suggested that these molecules could not fit into the auxin-binding pocket of the TIR1. It has been shown experimentally that these molecules fail to interfere with auxin perception, Aux/IAA degradation, and the downstream auxin signaling pathway (Tsuda et al., 2011). In contrast, alkoxy-auxins block the auxin transport activity of the PIN, ABCB, and AUX1 transporters in both yeast assays and *in planta*. Therefore, alkoxy-auxins are meant to become important tools to uncouple perception and transport in complex auxin mediated processes (Tsuda et al., 2011; Ma and Robert, 2014).

Long distance transport has also been reported for several hormones, but the molecular mechanisms are just emerging. ABA, cytokinin, strigolactones and jasmonates were detected in phloem or xylem, suggesting that these molecules could either be actively extruded from the cell or simply cross membranes by diffusion into the vascular tissue (Thorpe et al., 2007; Kudo et al., 2010; Kohlen et al., 2011). As in the case of auxins, small molecules provide useful tools to analyse the transport of other hormones. For example, specific ABC transporters inhibitors such as glibenclamide, verapamil and vanadate have been used to confirm role of the proteins AtABCG25 and AtABCG40 as ABA transporters (Kuromori et al., 2010; Kang et al., 2010).

New evidence exists that gibberellins too are actively transported; Shani and colleagues (2013) synthesized fluorescein labeled GA molecules (GA4- and GA3-fluorescein) that could be visualized in root cells and preferentially accumulate in the endodermal cells (Figure 1, Table 1 and Supplemental Table 1). By using mitochondrial ATP synthesis inhibitors such as antimycin A, oligomycin A and myxothiazol, the researchers demonstrated the specific GA accumulation in the endodermis relies on active, energy dependent mechanisms, suggesting an active GA transport (Shani et al., 2013).

The idea of cytokinin-specific transporters is still an open question (Bishopp et al., 2011a). Podlešáková et al. (2012) generated a series of novel analogs of cytokinin and observed that some of these compounds had different transport affinities, hinting at the possibility of identifying immobile CK analogs. The structure-activity analysis of these immobile CK as well as the identification of their targets might help to define components of the CK transport system.

Wounding triggers systemic responses that depend on the *de novo* synthesis of JA and JA-Ile in distal leaves in *Arabidopsis* (Koo et al., 2009; Wasternack and Hause, 2013), whereas grafting experiments with mutants excluded systemic formation of JA in tomato (Li et al., 2002; Koo and Howe, 2009). In principle this advocates against the transport of JA or JA-Ile. However, using radioactively labeled molecules, Me-JA, JA and JA-Ile were all found in phloem and/or xylem (Baldwin and Zhang, 1997; Thorpe et al., 2007; Matsuura et al., 2012). In addition, a functional fluorescent-labeled jasmonate probe was reported to migrate in the vascular tissues of tomato plants (Liu et al., 2012; Liu and Sang, 2013). We envision that the development of fluorescent-labeled hormones combined with the use of chemicals inhibiting different transport mechanisms will be essential tools with which to address the transport of jasmonates (Rigal et al., 2014). Many hypotheses have been proposed to explain the nature of systemic wound signals, being electric signals a possibility, and recently glutamate receptor-like genes (GLR), similar to those involved in synaptic activity in animals, have been implicated (Mousavi et al., 2013). In addition, three GLR antagonists were identified through a pharmacological screen for molecules inhibiting the growth of tobacco pollen tubes. Furthermore, the analysis of the GLR agonistic amino acids showed that D-serine is the most active agonist promoting pollen tube growth. D-serine is secreted naturally by the pistil to mediate pollen tube guidance (Michard et al., 2011). As D-serine is a modulator of animal neuronal circuits, this finding shows an astonishing analogy between electrochemical signal transduction in plants and animals.

## Phytohormone perception

Phytohormones are active at very low concentrations due to their high-affinity recognition systems. Since perception is the first step for the activation of downstream signaling cascades, researchers have prioritized the identification hormone receptors and perception components. Although many components of the hormonal perception system were identified by classical genetic approaches, the use of phytohormone analogs and chemical genomics was important for the detailed dissection of the underlying molecular mechanisms through which they function. For example, NPA was instrumental in identifying several components of the auxin pathway. These include *TIR1*, the founder member of the auxin receptor family TIR1/AFB proteins (Ruegger et al., 1997; Mockaitis and Estelle, 2008).

Coronatine, the bacterial mimic of JA-Ile, was instrumental in the identification of the *coi1* (*coronatine insensitive 1*) mutant. It was subsequently discovered that *coi1* was impaired in the F-box component of the JA-Ile receptor (Xie et al., 1998; Sheard et al., 2010). In addition, a small-scale screen of oxylipins, JA precursors and derivatives identified the synthetic isomer (+)-JA-L-Ile as a strong jasmonate agonist (Fonseca et al., 2009b). The structure of coronatine and the synthetic (+)-JA-L-Ile suggests that the stereochemical orientation of the cyclopentanone-ring side chains greatly affects receptor binding. Purification of the two natural epimers demonstrated that pure (−)-JA-L-Ile is inactive and that the active hormone is (+)-*7-iso*-JA-L-Ile, which is structurally more similar to coronatine (Fonseca et al., 2009b). Besides, the activity of COI1 as the JA-Ile receptor was first demonstrated by using radiolabeled coronatine in competitive binding assays (Katsir et al., 2008). To assess the direct binding of jasmonates to the COI1 receptor, biotin-tagged photoaffinity probes of JAs were designed (Yan et al., 2009a). The coronatine photoaffinity probe (PACOR), which retained weak biological activities, physically binds with the purified COI1 protein, further supporting that COI1 directly binds to COR and serves as a receptor for jasmonate (Yan et al., 2009a). All of these results show clearly the importance of JA-Ile analogs in several of the most important advances in phytohormone research.

### The redundancy/specificity paradox of hormone receptors

Chemical genomic studies can also be used to address the striking receptor redundancy/specificity paradox. Many components of hormone receptor complexes belong to large gene families and are functionally redundant. For example, the *Arabidopsis* genome encodes 14 *PYR/PYL* genes and 12 *JAZ* genes (Chini et al., 2007; Thines et al., 2007; Park et al., 2009). Although members of these families regulate the same hormone-mediated responses, individual members confer some tissue- and process-specificity.

The auxin perception complex shows the greatest redundancy of all the pathways discussed in this review. It is composed of one F-box member (among the 6 possible TIR1/AFB proteins), one co-receptor (among the 29 possible Aux/IAAs) alongside the single IP6 cofactor. The identification of auxin analogs has helped to address both the redundancy and specificity of various components within the auxin perception machinery. For example, mutations in the auxin receptor, AFB5, were identified in a genetic screen for lines resistant to the picolinate auxin (Walsh and Chang, 2006). *afb5* is highly resistant to picolinate auxins (such as picloram or DAS534) but not to other auxin isoforms such as 2,4-D or IAA (Table 1 and Supplemental Table 1). This suggests that picolinate is a highly specific agonist of the auxin pathway (Walsh and Chang, 2006). Interestingly exogenous application of picloram mimics some aspects of auxin responses that application of 2,4-D or IAA application fails to reproduce, such as hypocotyl elongation. Although TIR1 and AFB5 show an almost identical secondary structure, biochemical analyses show that the TIR1–IAA7 and AFB5–IAA7 co-receptor complexes exhibit different auxin-binding affinities (Figure 1). Indeed, a single amino acid substitution has been identified through docking analyses that is responsible for the change in affinities of TIR1 and AFB5 for IAA and picloram (Calderón Villalobos et al., 2012). These data demonstrate that the AFB5-Aux/IAA co-receptor selectively binds picloram, but not IAA, whereas TIR1-Aux/IAA accepts IAA, but not picloram, providing the first mechanistic explanation for specificity in auxin perception.

### From molecules to functions: the power of chemical genomics

Chemical genomics approaches have also been instrumental in the discovery of the redundant ABA receptors, as different compounds show specificity to certain groups of receptors. Pyrabactin was originally identified as a synthetic inhibitor of only one ABA-mediated response, seed germination (Zhao et al., 2007). A screen was performed for pyrabactin-resistant mutants aiming to identify redundant components of the ABA pathway (Cutler and McCourt, 2005). Indeed, single pyrabactin resistant mutants (*pyr*) were sensitive to ABA, whereas only multiple mutants in *PYR1*/*PYR1-like* (*PYL*) genes exhibited ABA insensitivity, demonstrating the functional redundancy of family members (Park et al., 2009). Additional studies using small molecules assessed the structural requirements of the binding pocket of the PYR/PYL receptors (Cao et al., 2013; Okamoto et al., 2013). For example, pyrabactin binds and activates two of the PYR/PYL receptors, while quinabactin activates three additional PYR/PYLs (Table 1 and Supplemental Table 1). Since pyrabactin affects ABA-related processes in seeds and quinabactin regulates ABA-dependent stomatal closure, these chemicals are shedding light on the partially redundant functions of the PYR/PYL ABA receptors (Figure 1).

In the case of cytokinin, a chemical genomic approach was employed to identify molecules antagonizing the activity of the cytokinin receptor CRE1 (CYTOKININ RESPONSE 1; Arata et al., 2010). The authors elegantly generated a yeast system based on the *Arabidopsis CRE1* gene conferring cytokinin dependent growth. This system allowed a high-throughput screen looking for growth defects in yeast grown in the presence of cytokinin, and identified two compounds (SS-6772 and S-4607) that inhibited the CRE1-dependent yeast growth (Table 1 and Supplemental Table 1). These compounds were chemically quite distinct from previous reported cytokinin receptor antagonists and new variations of these compounds were generated introducing minor modification of the quinazoline ring (Spíchal et al., 2009; Arata et al., 2010; Nisler et al., 2010). A new antagonist, S-4893, was confirmed as a strong inhibitor of cytokinin signaling in both yeast system and *in planta*. Further biochemical and genetic studies revealed that S-4893 acts as a non-competitive inhibitor of CRE1 not only in *Arabidopsis* but also in rice, suggesting that this compound operates in a range of plant species to antagonize cytokinin-mediated processes (Figure 1).

Perception of BR occurs at the plasma membrane by the receptor BRASSINOSTEROID INSENSITIVE (BRI1). In order to investigate endocytosis of the receptor-ligand complex, researchers developed a bioactive fluorescent labeled BR, called fluorescent castasterone (AFCS) (Irani et al., 2012) (Figure 1, Table 1 and Supplemental Table 1). They used this tool to show that trafficking and endocytosis of the BRI1-AFCS complex is dependent on clathrin, ARF GTPases and the Rab5 GTPase pathway. However, concanamycin A, a specific inhibitor of the trans-Golgi network/early endosome (TGN/EE) blocked the BRI1-AFCS complex at the TGN/EE without affecting the BR signaling. The integration of these chemical and genetic data showed that retention of active BRI1 at the plasma membrane, rather than in endosomes, is an important factor in activation of BR signaling.

The recent identification of many components of several phytohormone receptor complexes opens the opportunity to generate new molecular tools. Most plant co-receptor complexes are able to perceive their targets in heterologous systems such as yeast. For example, yeast two hybrid (Y2H) systems have been used to induce the formation of TIR1-Aux/IAA complex in an auxin-inducible manner (Calderón Villalobos et al., 2012) and in a similar way JA-Ile or COR promotes COI1-JAZ interaction in yeast (Fonseca et al., 2009a; Chini, 2014). As the hormone co-receptors are the only plant proteins expressed within these heterologous systems, they represent unique tools to identify small molecules directly perturbing the hormone perception. Compounds able to induce the formation of the perception complex can subsequently be used to identify novel active forms of the hormone. In contrast, compounds inhibiting the hormone-dependent co-receptor complex might be direct antagonist molecules.

### From receptor structures to molecules: rational design of phytohormone analogs

In the last decade, the crystal structures of several perception complexes were solved (Tan et al., 2007; Murase et al., 2008; Shimada et al., 2008; Park et al., 2009; Sheard et al., 2010). These structural data open new opportunities for the rational design of antagonist molecules specifically binding to and blocking the active pockets of individual receptors. The methodology of ligand-based rational design has been exploited extensively in medical research, but is just emerging in the agrochemical field (Lamberth et al., 2013). For example, this methodology has permitted the rational design of alfa-alkyl auxin molecules (Figure 1, Table 1 and Supplemental Table 1). These auxin analogs are able to specifically bind and block the formation of the hormone receptor complex was very successful and have allowed systematic structure-activity analysis of the alfa-position of IAA (Hayashi et al., 2008, 2012) (Figure 1). An advanced modification of one of these compounds generated the auxinole molecule (alfa-[2,4-dimethylphenylethyl-2-oxo]-IAA). This binds TIR1 strongly to block the formation of the TIR1-IAA-Aux/IAA receptor complex. Molecular docking studies have provided novel insights of the molecular mechanism of auxinole activity, predicting that auxinole strongly interacts with the Phe82 residue. This residue of TIR1 that is crucial for Aux/IAA recognition and blocks TIR1 activity by interacting with this critical amino acid. Hayashi et al. showed that auxinole and alfa-alkyl auxin molecules retain their antagonistic activity in crop plants such as tomato as well as in distant relatives, such as the moss *Physcomitrella patens* (Hayashi et al., 2008, 2012).

The same principle of rational design used around the crystal structure of the COI1/JAZ co-receptor to design a COR-derivative that binds to COI1 but spatially impedes the interaction of the COI-JAZ co-receptors (Figure 1). This compound, COR *O*-methyloxime (COR-MO), shows a strong activity inhibiting the formation of the COI1-JAZ perception complex and preventing JAZ degradation (Monte et al., 2014). COR-MO reverses the effects induced by exogenous JA-Ile or COR treatments on several JA-mediated responses efficiently, thereby underpinning its usefulness in dissecting the JA-pathway (Table 1 and Supplemental Table 1). Moreover, COR-MO enhances plant defense by preventing the effectiveness of the bacterial effector COR during *Pseudomonas syringae* infections. As this compounds works in a variety of different plant species, it further highlights the potential of such compounds in biotechnological and agronomical processes (Monte et al., 2014) (Figure 1).

In contrast to JA-Ile and auxins, which act as molecular glues by holding receptor complexes together, ABA binds within a cavity in its receptor where it induces conformational changes that in turn promote the interaction with the active site of group-A PROTEIN PHOSPHATASE 2C (PP2Cs) (Melcher et al., 2010). Following the resolution of the crystal structure of several ABA/PYR/PP2C complexes, Takeuchi and colleagues designed a series of ABA analogs (AS*n*) with long alkyl chains of the ABA 3′ ring CH, that they predicted would spatially block the PYL-PP2C interaction (Table 1 and Supplemental Table 1). A six-carbon alkyl chain was sufficient to produce a potent ABA antagonist able to block multiple ABA-mediated responses *in vivo* such as germination, the expression of known downstream response genes and PP2A activity (Takeuchi et al., 2014) (Figure 1).

Brassinolide (BL) is a potent brassinosteroid that binds the BR receptor BRI1 directly and induces the interaction between BRI1 and SERK1 (SOMATIC EMBRYOGENESIS RECEPTOR-LIKE KINASE1; Santiago et al., 2013). Based on the crystal structure of the BRI1-BL complex Muto and colleagues generated a alkylated version of BL called brassinolide-2,3-acetonide. This compound was able to bind BRI1 but sterically interferes with the SRRK1 interaction (Muto and Todoroki, 2013) (Figure 1, Table 1). Indeed, brassinolide-2,3-acetonide showed a clear BL antagonistic effect in rice seedlings and opens the opportunity to develop a set of chemical tools modulating BR perception and further dissect the BR response pathway.

Collectively, these examples of antagonist molecules highlight the usefulness of the structure-based design of hormone analogs specifically binding for and blocking the active pocket of the receptors. This approach provides a novel methodology for generating bioactive hormone analogs.

## Specificity and redundancy

Another important contribution of chemical genomic screens is the possibility to assess specificity within signaling pathways or specific developmental processes. Essentially this notion is based on the fact that chemicals can overcome functional redundancy by inhibiting multiple members of a redundant protein family (Cutler and McCourt, 2005). A good example described earlier is pyrabactin, a compound affecting a single ABA-mediated response, germination (Zhao et al., 2007). The analyses of the first pyrabactin resistant (*pyr*) and further *pyr/pyl* mutants revealed nicely the functional redundancy of the 14-member PYR/PYL family for multiple ABA responses (Park et al., 2009).

Bikinin was identified in a screen for molecules inducing constitutive BR-related phenotypes such as hypocotyl elongation, petiole elongation and pale, blade shaped leaves (De Rybel et al., 2009). Strikingly, bikinin induces BR responses in mutants deficient in BR biosynthesis, perception and signaling. Bikinin also stimulates BR responses in *bin2-1*, a gain of function mutation in BIN2 (BRASSINOSTEROID-INSENSITIVE2). BIN2 is a GSK3 (GLYCOGEN SYNTHASE KINASE3) kinase that phosphorylates and inactivates the key transcription factors in the BR pathway, BZR1 and BES1/BZR2 (He et al., 2002). Bikinin acts as a competitive inhibitor of ATP binding and binds BIN2 directly causing the inhibition of seven of the 10 GSK3 kinases (Vert and Chory, 2006; De Rybel et al., 2009; Yan et al., 2009b). One bikinin-inhibited GSK3 kinases, ASK θ, interacts directly with and phosphorylates BEH2 (BES1/BZR1 HOMOLOG 2), a BR responsive transcription factor closely related to BZR1 and BES1/BZR2 (Yin et al., 2005b; Rozhon et al., 2010). Therefore, the discovery of bikinin allowed the identification of new components of the BR pathway and also enabled the conditional blockage of multiple key regulators in BR signaling, providing an essential tool to study the BR regulatory mechanisms.

Bestatin is an inhibitor of aminopeptidase and powerful inducer of JA- and wound-response genes in tomato (Schaller et al., 1995). The root growth inhibitory effect of bestatin depends on the key transcription factor of the JA pathway MYC2 but seems independent of the JA-Ile receptor COI1 (Figure 1). Therefore, Zheng et al. (2006) used bestatin to identify new components of the wounding signaling pathway dependent on JA-Ile and MYC2. Several bestatin resistant mutants (*ber*) were isolated, some of which allelic to *jin1/myc2*. In addition, *ber6* carries a mutation in *MED25/PFT1* (*MEDIATOR 25/PHYTOCHROME AND FLOWERING TIME 1*). This gene encodes for a subunit of the eukaryotic transcription mediator system (Zheng et al., 2006). MED25/PFT1 was first described as a positive regulator of shade avoidance and has subsequently been shown to also be required for plant defense (Cerdán and Chory, 2003; Kidd et al., 2009). Recent studies showed that MYC2, MYC3 and MYC4 have redundant roles in plant defense; MED25 directly interacts with MYC2 and it is required for MYC2-dependent pathogen defense (Fernández-Calvo et al., 2011; Çevik et al., 2012; Chen et al., 2012; Schweizer et al., 2013). MYC2, MYC3, and MYC4 are regulated by light quality and are involved in shade avoidance responses (Robson et al., 2010; Chico et al., 2014). Therefore, the use of bestatin to isolate mutants in *MED25/PFT1* suggested the redundant role of the MYC2, MYC3, and MYC4 in defense and shade avoidance responses. The use of bestatin can potentially identify new regulators of the MYCs and help to assess redundancy.

Strigolactones have long been studied because of their importance in stimulating the growth of the parasitic *Striga* and *Orobanche* on several crops. Structure-activity relationship analyses showed that several analogs mimic strigolactone functions (reviewed by Janssen and Snowden, 2012). Different structural requirements regulate strigolactone-mediated processes such as seed germination, hyphal branching of arbuscular mycorrizal fungi and shoot branching inhibition (Kondo et al., 2007; Zwanenburg et al., 2009; Akiyama et al., 2010; Fukui et al., 2011, 2013; Boyer et al., 2012; reviewed by Zwanenburg and Pospísil, 2013). Furthermore, newly synthesized strigolactone competitive analogs suggest that *Arabidopsis*, Orobanche and arbuscular mychorrial fungi possess variations in the sensitivity to strigolactone analogs, providing additional support to the idea that variations in strigolactone receptors among the different species should exist (Cohen et al., 2013; reviewed by Janssen and Snowden, 2012).

Karrikins are compounds structurally similar to strigolactones. They promote germination, but unlike strigolactones, karrikins are not produced in plants, but instead are found in the smoke of burning plant material. Despite this, in many ways they behave as hormones, as small quantities of the signal is sufficient to trigger a signal transduction pathway. Genetic screenings for karrikin insensitive mutants showed that karrikins perception share a common mechanism with strigolactones. The F-box MAX2 (MORE AXILLARY BRANCHES 2) is required to perceive both kinds of compounds. In both cases an alfa/beta hydrolase fold protein [KAI2 (KARRIKIN INSENSITIVE 2) or DAD2/D14] is part of the receptor complex (Nelson et al., 2011; Hamiaux et al., 2012; Waters et al., 2012; Guo et al., 2013). The strigolactone and karrikin pathways are a good example of how structurally similar molecules rely on similar—or even common—perception mechanisms and confer overlapping physiological responses while maintaining their identity in terms of structure-function (Figure 1, Table 1 and Supplemental Table 1).

## Phytohormone crosstalk

It has been well documented that most biological processes are not regulated by a single hormone but rather by complex signaling networks controlled by multiple hormones or other signaling components. For example, auxin and cytokinin act in consort to control the formation of the embryonic root, root meristem size, root branching, vascular pattern and shoot phylotaxy (Dello Ioio et al., 2008; Bishopp et al., 2011b; Besnard et al., 2014). Chemical genomic approaches not only facilitate the dissection of hormonal pathways but could also shed light on the non-linear networks within which they operate as well as identify new downstream biological functions. For example, screening for compounds that affect gravitropism led to the identification of multiple chemicals that affect membrane trafficking in auxin dependent and independent manners (Surpin et al., 2005). Such important and sometimes unexpected results demonstrate the power of screens for small molecules regulating related biological processes.

Recently, a chemical genomic screen for molecules perturbing germination identified cotylimides, a class of compounds structurally similar to strigolactones that recapitulate cotyledon bleaching promoted by GR24 (Tsuchiya et al., 2010). The subsequent screen for mutants insensitive to cotylimides isolated several suppressor lines showing elongated hypocotyls, a phenotype commonly observed in mutants defective in light signaling. The analysis of strigolactones in light responses showed that these compounds mimic light in seedling growth and increase the accumulation of HY5 (ELONGATED HYPOCOTYL5), a protein directly targeted by COP1 (CONSTITUTIVELY PHOTOMORPHOGENIC 1) for degradation. Tsuchiya and colleagues elegantly revealed the molecular mechanism behind this response. Strigolactones mediate the nuclear exclusion of COP1, this stabilizes HY5, which in turn reduces hypocotyl elongation.

As described earlier in this review, L-kynurenine was identified in a chemical genomic screen. This compound was instrumental in showing that auxin induces the nuclear accumulation of the key activator of the ET pathway, EIN3 (ETHYLENE-INSENSITIVE3). Kyn was used to unravel a positive feedback loop between auxin biosynthesis and ET signaling (He et al., 2011), that was not detectable by conventional genetic analysis.

Brassinopride (BRP) was identified in a screen for inhibitors of brassinosteroids, based on the inhibition of BR-mediated hypocotyl growth in the dark and activation of the BR-responsive reporter gene *CPD:GUS* (Gendron et al., 2008) (Figure 1). The site of action of BRP has not been defined yet, however, application of brassinolide can reverse BR related effects of BRP. This suggests that BRP could perturb BR biosynthesis. Unexpectedly, BRP also induced exaggerated formation of apical hooks, resembling plant subjected to ET treatments. Additionally, the apical hook phenotype could be blocked by a chemical inhibitor of ethylene perception or by ET insensitive mutants, suggesting that BRP activates ET responses. Phenotypic analysis of ET and BR mutants in combination with analysis of the effects of BRP analogs, revealed a crosstalk between ET and BR in etiolated seedlings. Moreover, variation among BRP analogs suggest that modifying the side groups of BRP can have specific effects on BR or ET functions (Gendron et al., 2008).

Recently, it has also been proposed that hormone derivates can interfere with different signaling pathways (Katsir et al., 2008; Staswick, 2009; Gutierrez et al., 2012). For example, hormone conjugation is a common process in plants to activate, store or deactivate phytohormones, however, some conjugates show unexpected activity. When combined with either auxin or JA, tryptophan (Trp) conjugates with indole-3-acetic acid (IAA-Trp) or with jasmonic acid (JA-Trp). These conjugates act as an inhibitors of auxin responses, preventing several auxin-mediated responses, such as gravitropism, lateral root production and expression of known auxin response genes (Staswick, 2009). The evidence that an endogenous JA derivate inhibits the auxin pathway adds a novel level of regulation between the jasmonate and auxin pathways. It was hypothesized that IAA-Trp and JA-Trp could directly interfere with the auxin receptor TIR1-IAA/Aux as the TIR1 is required for IAA-Trp and JA-Trp inhibition and because these conjugated compounds are structurally similar to the active forms. However, IAA-Trp and JA-Trp do not directly alter the IAA-dependent interaction between TIR1 and Aux/IAA7 and their precise mode of action remains unknown (Staswick, 2009).

## Future prospectives

Plant chemical biology has enabled significant advances in phytohormone research. In addition to the classical phytohormone analogs widely used by the scientific community, we have recently witnessed the extensive use of chemical genomic approaches. The straightforward availability of large, diverse chemical libraries and the natural chemical resources will surely facilitate a further extension of this methodology and the identification of novel compounds regulating many biological processes of hormone synthesis, transport and response. Complementary, rational design of novel molecules and molecule labeling are emerging but successful strategies.

Therefore, we envision the continued use of plant chemical biology not only to identify novel components or regulation mechanisms of phytohormone pathways but also to better understand their mode of action and molecular networks. The discovery of molecules with certain specificity constrains will certainly contribute toward more rational and sustainable agriculture systems.

### Conflict of interest statement

The authors declare that the research was conducted in the absence of any commercial or financial relationships that could be construed as a potential conflict of interest.
