# Supplementary material for: Molecular locks and keys: the role of small molecules in phytohormone research
Source: Front Plant Sci. 2014 Dec 17;5:709. doi: 10.3389/fpls.2014.00709 (PMC4269113; doi:10.3389/fpls.2014.00709)
Supplement: Supplementary file 1 [file Table1.PDF]

**Supplemental Table I** – Resume of the relevant compounds mentioned in the text ordered by, the hormone they are related to, the biological process in which they interfere, and the corresponding nature (endogenous or synthetic) and mode of action (agonist, antagonist, inhibitor). In each box from top to the bottom: abbreviated name, structure, target and reference. **(1)** Among the Alkyl-auxins, the one represented is *tert*-butoxycarbonylaminohexyl-indole-3-acetic acid **(2)** among the Alcoxy-auxins, the one represented is BZ-IAA (5-benzyloxyindole-3-acetic acid).
Abreviations: IAA, indole-3-acetic acid.; NAA, 1-naphtaleneacetic acid; 2,4-D, dichlorophenoxyacetic acid; IAA-Trp, indole-3-acetyl-tryptophan.; JA-Trp, jasmonoyl-L-tryptophan; BUM, 2-[4-(Diethylamino)-2-hydroxybenzoyl]benzoic acid; GA3, gibberellic acid3; GA4, gibberellic acid4; Phe-Ade, N-Phenyladenine; AS6, 3′-alkylsulfanyl ABA6 (3'α-*n*-hexylthio-abscisic acid); Cor-MO, coronatine-*O*-methyloxime; PACOR, coronatine photoaffinityprobe; BTH, benzothiadiazole; INA, 2,6-dichloroisonicotinic acid.

| AUXIN | | | | | | | | |
|---|---|---|---|---|---|---|---|---|
| | Endogenous | Synthetic agonists | | | Antagonists | | | |
| PERCEPTION | **IAA**<br>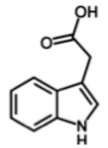<br>TIR1/AFBs | **NAA**<br>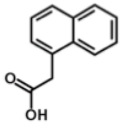<br>TIR1/AFBs | **2,4-D**<br>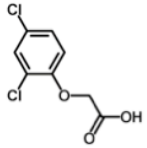<br>TIR1/AFBs | **Picloram**<br>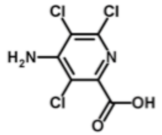<br>AFB5<br>Walsh et al., 2006<br>Villalobos et al., 2012 | **Auxinole**<br>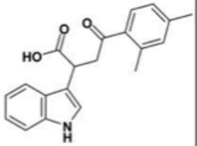<br>TIR1/AFBs<br>Hayashi et al., 2012 | **Alkyl-auxins (1)**<br>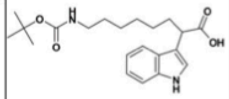<br>TIR1<br>Hayashi et al., 2008 | **IAA-Trp**<br>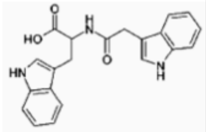<br>Unknown target<br>Staswick et al.,2009 | **JA-Trp**<br>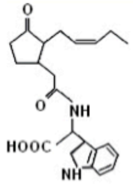<br>Unknown target<br>Staswick et al., 2009 |

| | Inhibitors | | | Inhibitors | | |
|---|---|---|---|---|---|---|
| BIOSYNTHESIS | **L- kynurenine**<br>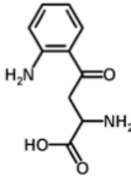<br>TAA1/TARs<br>He et al., 2011 | | TRANSPORT | **BUM**<br>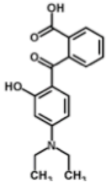<br>ABCB/MBR/PGP efflux carriers<br>Kim et al., 2010 | **Alcoxy-auxins (2)**<br>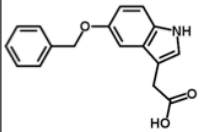<br>Auxin transporters PIN, ABCB and AUX<br>Tsuda et al., 2011 | **Gravacin**<br>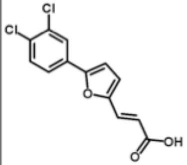<br>PGP19<br>Rojas-Pierce et al., 2007 |

| GIBBERELIN | | | |
|---|---|---|---|
| | Endogenous | | Synthetic agonists |
| PERCEPTION | **GA3**<br>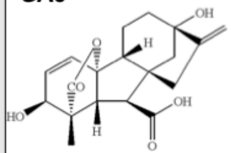<br>GID1 receptor | **GA4**<br>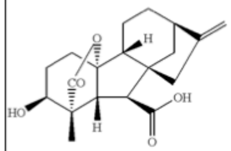<br>GID1 receptor | **GA3 –Fluorescein**<br>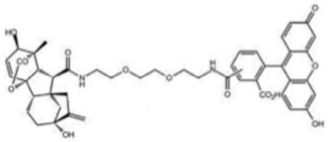<br>GID1 receptor<br>Shani et al., 2013 |

| CYTOKININ | | | | | | |
|---|---|---|---|---|---|---|
| | **Endogenous** | | **Synthetic antagonists** | | | |
| **PERCEPTION** | **Kinetin** | ***trans*-Zeatin** | **Phe-Ade** | **S-4893** | **SS-6772** | **S-4607** |
| | 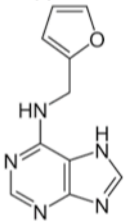 | 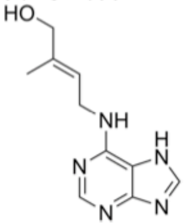 | 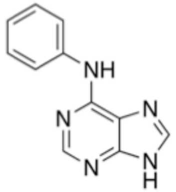 | 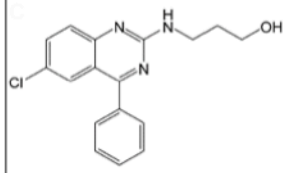 | 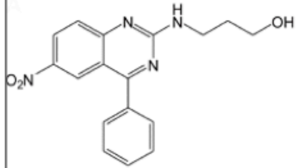 | 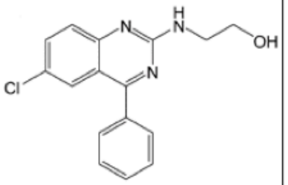 |
| | CRE1 receptor | CRE1 receptor | CKX, AHK3 and CRE1/AHK4<br>Motte et al., 2013 | CRE1 receptor<br>Arata et al., 2010 | CRE1 receptor<br>Arata et al., 2010 | CRE1 receptor<br>Arata et al., 2010 |

| JASMONIC ACID | | | | | | |
|---|---|---|---|---|---|---|
| | **Endogenous** | **Natural agonist (*Pseudomonas*)** | **Synthetic agonists** | | | **Synthetic antagonist** |
| **PERCEPTION** | **(+)-*7-iso*-JA-L- Ile** | **Coronatine** | **(+)-JA-Ile** | **PACOR** | **Fluorescent jasmonate** | **Cor-MO** |
| | 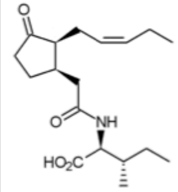 | 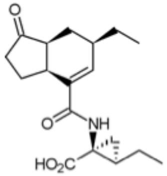 | 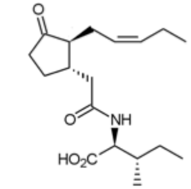 | 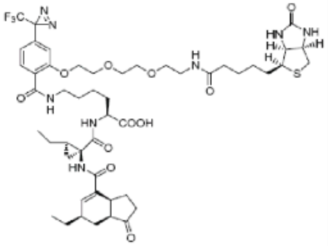 | 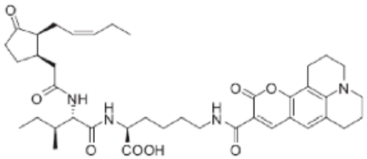 | 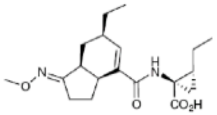 |
| | COI1/JAZs<br>Fonseca et al., 2009 | COI1/JAZs<br>Xie et al., 1998 | COI1/JAZs<br>Fonseca et al., 2009 | COI1/JAZ1<br>Gu et al., 2010 | Unknown target<br>Liu and Sang, 2013. | COI1/JAZs<br>Monte et al., 2014 |

| | | | | |
|---|---|---|---|---|
| | **Endogenous inhibitors** | **Synthetic inhibitors** | | |
| **BIOSYNTHESIS** | **Vernolic Acid** | **Phenidone** | **Jarin-1** | **JM-8686** |
| | 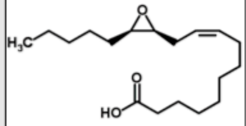 | 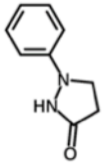 | 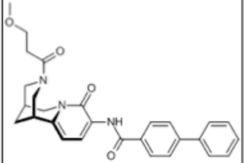 | 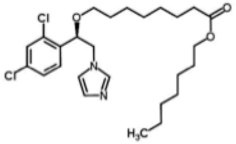 |
| | AOC2<br>Hofmann et al., 2006 | LOX2<br>Engelberth et al.,2011 | JAR1<br>Meesters et al., 2014 | AOS<br>Oh et al., 2006 |

| | |
|---|---|
| | **Signal activators** |
| **SIGNALING** | **Bestatin** |
| | 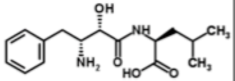 |
| | Aminopeptidases<br>Schaller et al.,1995<br>Zheng et al., 2006 |

| ABA | | | | |
|---|---|---|---|---|
| | **Endogenous** | **Synthetic agonists** | | **Antagonists** |
| **PERCEPTION** | **(+)-Abscisic acid**<br>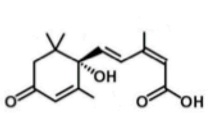<br>PYR/PYL receptors | **Pyrabactin**<br>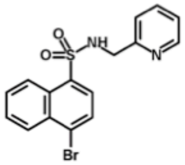<br>PYR1 and PYL1<br>Park et al., 2009<br>Okamoto et al., 2013 | **Quinabactin**<br>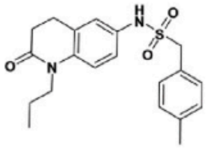<br>PYR1, PYL1-3,4<br>Okamoto et al., 2013 | **AS*n* (AS6)**<br>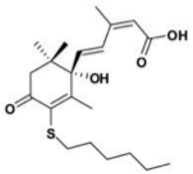<br>PYR/PYL receptors<br>Takeuchi et al., 2014 |

| BRASSINOSTEROID | | | | | | | |
|---|---|---|---|---|---|---|---|
| | **Endogenous** | **Synthetic analogs** | | **Synthetic inhibitor** | | **Synthetic inhibitors** | |
| **PERCEPTION** | **Brassinolide**<br>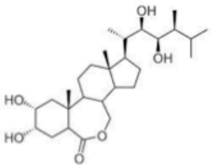<br>BRI1 receptor | **Fluorescent Castasterone**<br>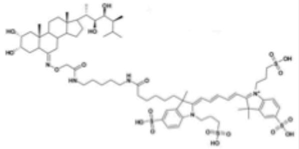<br>BRI1<br>Irani et al., 2012 | **SIGNALING** | **Bikinin**<br>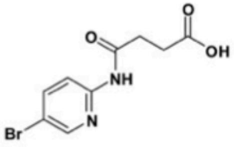<br>GSK3 kinases<br>De Rybel et al., 2009 | **BIOSYNTHESIS** | **Brassinazole**<br>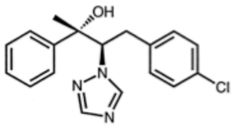<br>Cytochromes P450<br>DWF4 and CPD<br>Asami et al., 2000<br>Asami et al, 2001 | **Brassinopride**<br>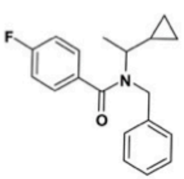<br>Unknown target<br>Gendron et al., 2008 |

| STRIGOLACTONES | | | | |
|---|---|---|---|---|
| | **Endogenous** | **Synthetic analogs** | | **Natural mimetics (from smoke)** |
| **PERCEPTION** | **(+)-strigol**<br>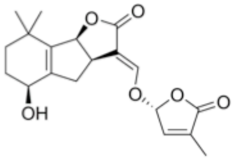<br>MAX2/DAD2/D14 | **GR24**<br>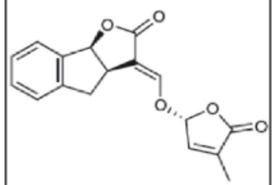<br>MAX2/DAD2/D14<br>Gomez-Roldan et al. 2008<br>Umehara et al., 2008 | **Cotylimides(CTL-VI)**<br>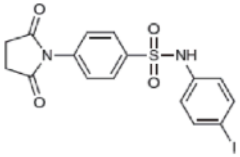<br>Unknown targets<br>Tsuchyia et al., 2010 | **Karrikins (KAR2)**<br>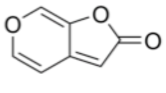<br>MAX2/KAI2<br>Nelson et al., 2011<br>Harmiaux et al., 2012<br>Waters et al., 2012 |

| SALICYLIC ACID | | | | | | |
|---|---|---|---|---|---|---|
| PERCEPTION | Endogenous | Synthetic analogs | | | SYNTHESIS/ CATABOLISM | Synthetic inhibitors |
| | **SA** 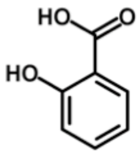  NPR receptors | **BTH** 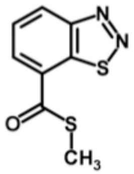  Unknown targets Gorlach et al., 1996 Lawton et al., 1996 | **INA** 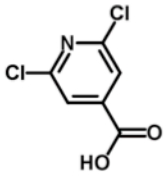  Unknown targets Conrath et al., 1995 | | | **Imprimatins (C2)** 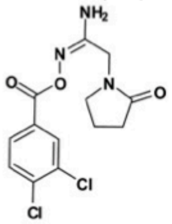  SA glucosyltransferases Noutoshi et al., 2012 |
